# Investigating the Effect of Tube Diameter on the Performance of a Hybrid Photovoltaic–Thermal System Based on Phase Change Materials and Nanofluids

**DOI:** 10.3390/ma15217613

**Published:** 2022-10-29

**Authors:** Saeed Alqaed, Jawed Mustafa, Fahad Awjah Almehmadi, Mathkar A. Alharthi, Mohsen Sharifpur, Goshtasp Cheraghian

**Affiliations:** 1 Mechanical Engineering Department, College of Engineering, Najran University, P.O. Box 1988, Najran 61441, Saudi Arabia; 2 Department of Applied Mechanical Engineering, College of Applied Engineering, Muzahimiyah Branch, King Saud University, P.O. Box 800, Riyadh 11421, Saudi Arabia; 3 Department of Chemical Engineering, College of Engineering at Yanbu, Taibah University, Yanbu Al-Bahr 41911, Saudi Arabia; 4 Department of Mechanical and Aeronautical Engineering, University of Pretoria, Private Bag X20, Hatfield 0028, Pretoria 0002, South Africa; 5 Department of Medical Research, China Medical University Hospital, China Medical University, Taichung 404, Taiwan; 6 Institut für Chemie and IRIS Adlershof, Humboldt-Universität zu Berlin, Germany; 7 Department of Chemistry, King’s College London, London, UK

**Keywords:** two-phase organic nanofluid, solar energy, environment, organic PCM

## Abstract

The finite element (FEM) approach is used in this study to model the laminar flow of an eco-friendly nanofluid (NF) within three pipes in a solar system. A solar panel and a supporting phase change material (PCM) that three pipelines flowed through made up the solar system. An organic, eco-friendly PCM was employed. Several fins were used on the pipes, and the NF temperature and panel temperature were measured at different flow rates. To model the NF flow, a two-phase mixture was used. As a direct consequence of the flow rate being raised by a factor of two, the maximum temperature of the panel dropped by 1.85 °C, and the average temperature dropped by 1.82 °C. As the flow rate increased, the temperature of the output flow dropped by up to 2 °C. At flow rates ranging from low to medium to high, the PCM melted completely in a short amount of time; however, at high flow rates, a portion of the PCM remained non-melted surrounding the pipes. An increase in the NF flow rate had a variable effect on the heat transfer (HTR) coefficient.

## 1. Introduction

Energy is vital to industrial activities and comfort, and humans have long been attempting to find new energy resources [1,2]. In recent years, scientists have worked to find new sources of energy. For long-term energy supply, new energy sources should be dependable and sustainable. Future energy supply might significantly benefit from solar energy [3]. It is a free and sustainable resource. An energy resource should inflict minimal damage on the environment [4,5,6]. The pollutants released by fossil fuels have been a major concern. For a better future, it is necessary to reduce environmental pollution [7,8,9]. Solar energy is clean and strongly contributes to environmental protection [10]. Numerous studies have been conducted on solar energy systems [11,12]. Bahaidarah et al. [13] evaluated a photovoltaic thermal (PVT) system in outdoor settings in the climate of Dhahran, Saudi Arabia, in February. They used water under the PVT panel to discharge the heat and reported a PVT module temperature reduction of 34%. NFs have been extensively used in heat exchange systems in recent years. They improve heat transfer (HTR) and raise system efficiency. NFs have been of great interest to researchers for solar energy systems. Khanjari et al. [14] studied the use of NFs in cold water for a PVT panel. They used a pipe and an absorptive surface and analyzed Ag/water and Al/water NFs. It was demonstrated that a rise in the nanoparticle volume fraction and input velocity increased HTR and efficiency. At a volume fraction of 5%, efficiency was calculated to be 8–10% for the Al/water NFs and 28–45% for the Ag/water NFs. 

The limited operation time due to limited sunshine duration is a major disadvantage of solar systems [15,16]. Solar energy storage has the potential to significantly increase the effectiveness of solar systems [17,18]. Electrical energy is stored in batteries throughout the day and supplied at night [19,20,21,22,23,24]. The ability to store thermal energy is also possible with phase change materials (PCM) [25,26,27,28]. In the literature on solar energy systems, PCMs have attracted the attention of academics recently [29,30,31]. Anib [32] experimentally investigated the performance of a solar air heater with a PCM and natural convection. The system consisted of air heating collectors and an operating cavity. It was employed to dry crops, medicinal plants, and livestock fodders. To dry medicinal plants, the hot air must be at a particular temperature; however, the temperature is destabilized in the absence of sunshine (heat source). Therefore, energy storage using a PCM helps keep the drying air at an almost stabilized temperature. 

Today, renewable energy resources, particularly solar energy, are an appealing topic for researchers. As fossil fuels are limited, new energy resources are required, and researchers seek to more effectively harvest solar energy [33,34,35]. The solar energy literature is extensive and ranges from solar power plants to small-scale solar residential water heaters [36,37,38]. The use of PCMs to store thermal energy is an effective method to supply heat in solar energy systems over periods when solar radiation is unavailable [39,40,41,42]. Furthermore, numerous researchers recommended NFs and lubricant in heat exchange systems to improve HTR [43,44,45,46,47,48]. In the present study, we evaluated the temperature of a solar system with a PCM. Three pipes through which an organic, eco-friendly NF flowed were employed within the PCM. The system was simulated for a period of 100 min at different flow rates to evaluate the temperature of the solar system. The organic PCM was employed to implement an eco-friendly solar system. The melted PCM volume fraction, solar panel temperature, output temperature, and HTR coefficient were measured at different flow rates. The novelty of the present work lies in the geometry of the solar system, PCM type, and the two-phase NF model.

**Figure 1:**
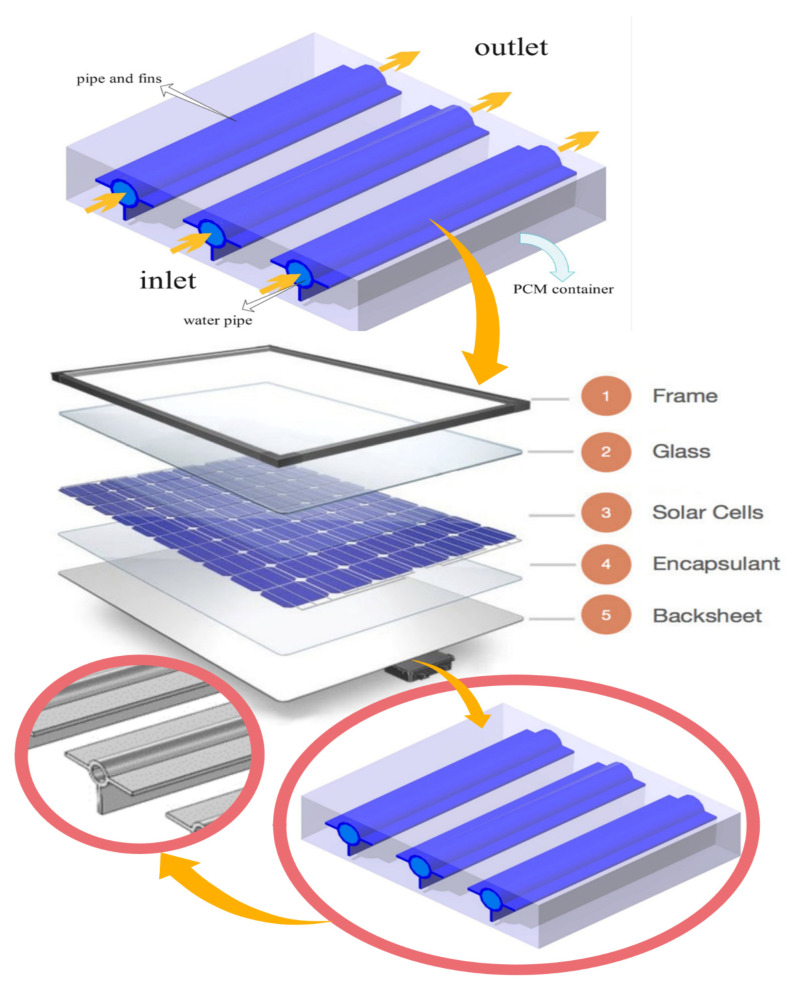
Schematic of the proposed photovoltaic–thermal hybrid system.

The proposed system consisted of a solar panel with several underlying pipes with a diameter of 6 cm surrounded by the organic, eco-friendly PCM, as shown in Figure 1. Table 1 reports the properties of the PCM. The organic NF flowed through the pipes in a laminar regime at a velocity of 0.1–0.2 cm/s. Table 2 provides the nanoparticle properties. The input velocity was fixed, and the output pressure was known. The no-slip boundary condition was applied to the pipe wall, and a constant heat flux of 850 W/m^2^ was applied to the panel. A volume percentage of 1% was used to prevent the sedimentation of nanoparticles. Hence, the sedimentation and accumulation of nanoparticles can be ignored in the calculations, and the amount of volume fraction can be enhanced up to 5%, according to Ref. [49]. More information on nanofluids can be found in Ref. [49].

## 2. Governing Equations

The governing equations of PCM are given, including mass, momentum, and energy conservation.

(1) $$\frac{\partial \rho }{\partial t}+\nabla \cdot \left(\rho \vec{U}\right)=0$$

(2) $$\frac{\partial }{\partial t}(\rho \vec{U)}+\nabla \cdot \left(\rho \vec{U}\vec{U}\right)=-\vec{\nabla }p+\rho \vec{g}+\nabla \cdot \bar{\bar{\tau }}+\vec{F}$$

(3) $$\frac{\partial \left(\rho H\right)}{\partial t}+\nabla \cdot \left(\rho \vec{U}\;H\right)=\nabla \cdot \left(K\nabla H\right)+S$$

The value of β, which represents the value of phase-changed PCM, can be obtained by using the following equation. The heat capacity of PCM is also achieved by employing the following equation.

(4) $$\beta_{k}=\{\begin{array}{ccc} \beta =0 & \;\mathrm{if}\; & T<T_{\mathrm{solidus}\;}\\ \beta =1 & \;\mathrm{if}\; & T>T_{\mathrm{Liquidus}\;}\\ \beta =\frac{T-T_{\mathrm{solidus}\;}}{T_{\mathrm{Liquidus}\;}-T_{\mathrm{solidus}\;}} & \;\mathrm{if}\; & T_{\mathrm{Liquidus}\;}<T<T_{\mathrm{solidus}\;} \end{array}$$

(5) $$\Delta C_{P\left(System\right)}=C_{P\left(npcm\right)}+L\times D\left(T\right)$$

B is the Boltzmann constant ($1.38\times 10^{-23}\;J/K$). 

The NF was assumed to be a Newtonian, incompressible, and two-phase mixture [50]. The following equations provide the conservation of mass, conservation of momentum, and conservation of energy for the two-phase mixture [51].

(6) $$\nabla .\left(\rho_{m}{\vec{v}}_{m}\right)=0$$

(7) $$\nabla .\left(\rho_{m}{\vec{v}}_{m}.\nabla {\vec{v}}_{m}\right)=-\nabla P_{m}+\nabla .\left(\mu_{m}\nabla {\vec{v}}_{m}\right)-\rho_{m,i}\beta_{m,i}g\left(T-T_{i}\right)+\;\nabla .\left(\sum_{k=1}^{n}Fi_{k}\rho_{k}{\vec{v}}_{dr,k}{\vec{v}}_{dr,k}\right)$$

(8) $$\nabla .\left(\overset{n}{\underset{k=1}{\sum }}Fi_{k}\rho_{k}c_{p,k}{\vec{v}}_{k}T\right)=\nabla .\left(k_{m}\nabla T\right)$$

The following equations provide the velocity equations and properties for the two-phase mixture [51].

(9) $${\vec{v}}_{m}=\frac{\sum_{k=1}^{n}Fi_{k}\rho_{k}{\vec{v}}_{k}}{\rho_{m}}$$

(10) $$\rho_{m}=\overset{n}{\underset{k=1}{\sum }}Fi_{k}\rho_{k}$$

(11) $$\mu_{m}=\overset{n}{\underset{k=1}{\sum }}Fi_{k}\mu_{k}$$

Volume fraction:

(12) $$\nabla .\left(Fi_{p}\rho_{p}{\vec{v}}_{m}\right)=-\nabla .\left(Fi_{p}\rho_{p}{\vec{v}}_{dr,p}\right)$$

The velocity of nanoparticles in phase *k* is given by:

(13) $${\vec{v}}_{dr,k}={\vec{v}}_{pf}-\overset{n}{\underset{i=1}{\sum }}\frac{Fi_{k}\rho_{k}}{\rho_{m}}{\vec{v}}_{fk}$$

The slip velocity is defined as the difference between the nanoparticle velocity and the velocity of the fluid at the position of the nanoparticle in the absence of the nanoparticle:

(14) $${\vec{v}}_{pf}={\vec{v}}_{p}-{\vec{v}}_{f}$$

(15) $${\vec{v}}_{pf}=\frac{\rho_{p}d_{p}^{2}\left(\rho_{p}-\rho_{m}\right)}{18\mu_{f}f_{\mathrm{drag}}\rho_{p}}\vec{a}$$

(16) $$f_{\mathrm{drag}}=[\begin{array}{c} 1+0.15Re_{p}^{0.687},\;Re_{p}\leq 1000\\ 0.0183Re_{p}^{0.687},\;Re_{p}>1000 \end{array}$$

The gravitational acceleration is defined as:

(17) $$\vec{a}=\vec{g}-\left({\vec{v}}_{m}.\nabla \right){\vec{v}}_{m}$$

The thermal conductivity and viscosity correlations for this NF are given in the following equations [52]. The volume fraction considered for the NF is 1% in all cases.

(18) $$\frac{\mu_{nf}}{\mu_{bf}}=1-2.5\varphi$$

(19) $$\frac{k_{nf}}{k_{bf}}=0.981+0.00114\times T+30.661\times \varphi$$

## 3. Numerical Model, Grid Study, and Validation

**Figure 2:**
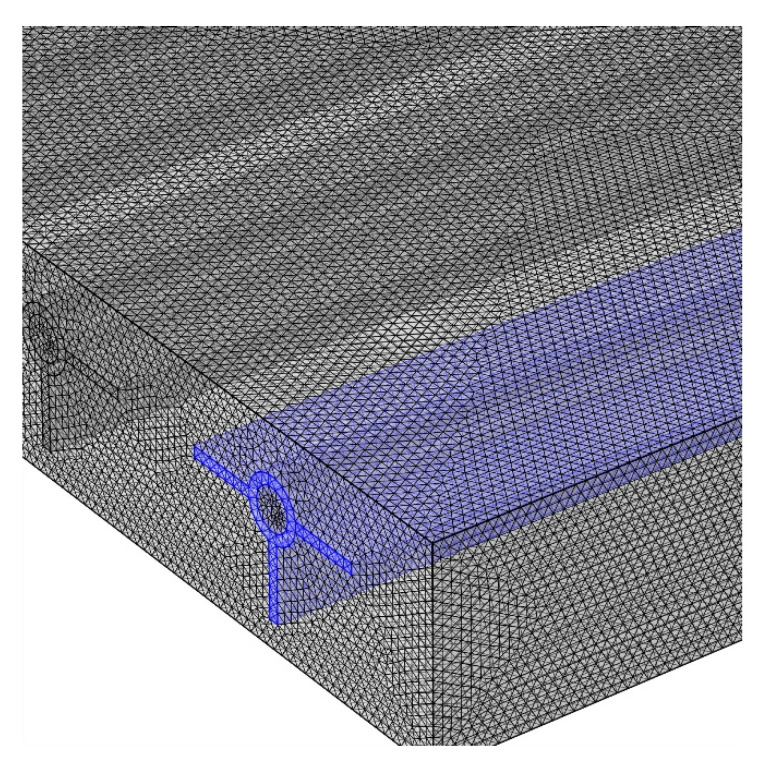
Meshed model of the panel and pipes.

The current research used a numerical methodology. The equations controlling the two-phase mixture flow and PCM were solved using the finite element method (FEM). There are two stages to the solution procedure for the FEM. In the beginning, the computational domain is divided into smaller domains. Each of these relatively limited domains stands for a different set of equations that apply to each constituent. All equations are solved simultaneously to perform the final calculations. This system of general equations can be solved using the initial values of the main problem. The NF entered the pipes at a constant velocity, and it exited the pipes at a constant pressure. The solar panel was exposed to a constant heat flow. Uneven meshes were used to mesh the computational domain, as seen in Figure 2.

To numerically simulate a model, it is required to develop the optimal grid to save time and cost. To identify the optimal grid, the average panel temperature was measured in different grids with different numbers of elements for a velocity of 0.1 cm/s. The grid with 1,144,853 elements was found to be the optimal grid to proceed with the simulations, as shown in Table 3.

To validate the numerical model, the numerical findings were compared to Bizhaem and Abbassi [53], who studied the forced convection of a two-phase NF within a pipe. Table 4 compares the numerical model and Bizhaem and Abbassi [53] in the pressure drop at the Reynolds numbers of 200, 500, and 1000 for volume fractions of 1% and 3%. The findings showed high agreement. 

For the second comparison, the present results are compared with experimental data prepared by Aghakhani et al. [54], who examined the outlet temperature values of water at different flow rates in a solar collector at different hours. In Table 5, the values of the outlet temperature of the water are compared with the results of Aghakhani et al. [54] for three flow rates. Due to the acceptable amounts of error, the accuracy of the simulation can be ensured.

## 4. Results and Discussion

**Figure 3:**
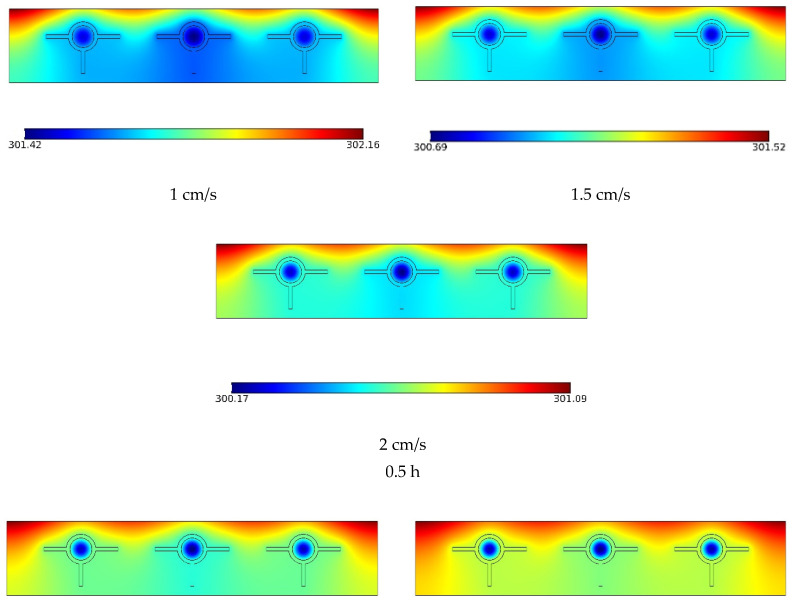

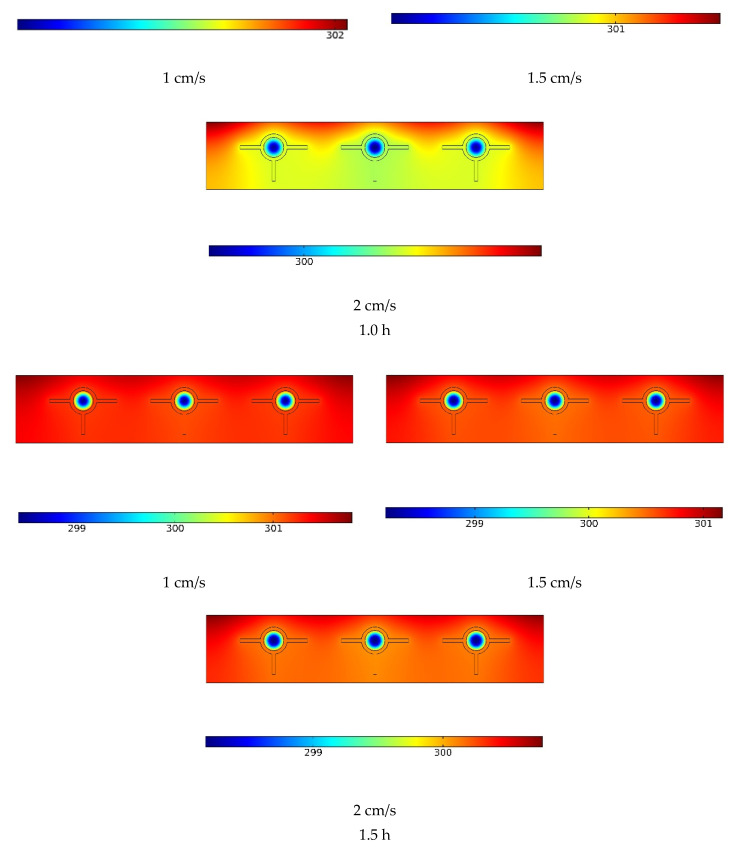
The temperature contour in the vertical middle section of the solar panel at three velocities and three times.

The central vertical segment of the solar panel’s temperature contour is shown in Figure 3 at three different velocities and three different times. The temperature rose with time, as can be observed. The NF and PCM had lower temperatures. However, the PCM melted over time, rising in temperature and leading to an increased flow temperature. The NF received heat that was transported from the top of the solar panel. Because of their lower HTR to the NF, the corners of the solar panel reached a greater temperature than the rest of the panel, especially at higher times [55,56]. As the flow rate rose, there was a corresponding rise in temperature throughout the solar panel.

**Figure 4:**
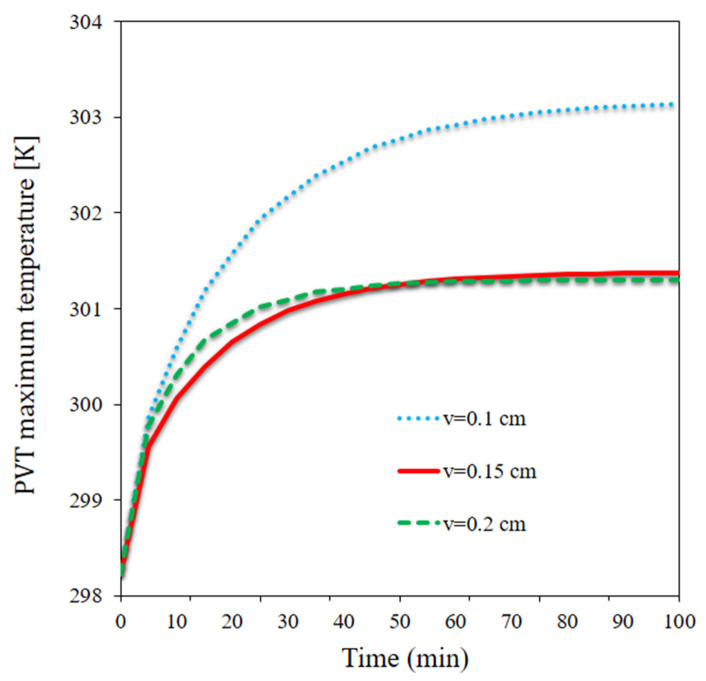
The maximum panel temperature at different velocities over time.

Figure 4 depicts the maximum panel temperature at different velocities over time. The maximum panel temperature dramatically rose from t = 0 to t = 10 min and then continued to rise at a smaller rate or even remained unchanged, depending on the flow rate. At lower flow rates, the heat discharge rate of the panel was lower. The discharge of heat through the NF was greater at higher flow rates, reducing the maximum panel temperature. A higher maximum panel temperature occurred at the lowest flow rate over time. However, the lowest maximum panel temperature was observed at the medium flow rate at times up to 50 min; at longer times (>50 min), the highest flow rate resulted in the highest maximum panel temperature. The maximum panel temperature was almost the same at the medium and high flow rates after 50 min. It was observed that the maximum panel temperature declined by 1.85 °C at t = 100 min as the flow rate doubled.

**Figure 5:**
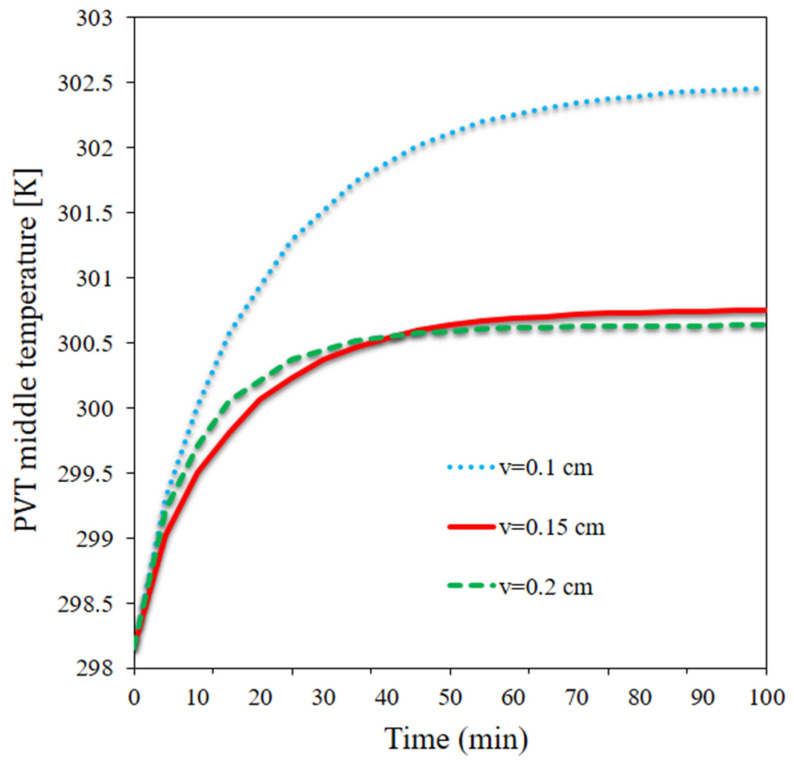
The average panel temperature at three velocities over time.

Figure 5 plots the average panel temperature at three velocities over time. The highest panel temperature and the average panel temperature almost followed the same pattern. It reduced by 1.82 °C as the flow rate doubled at t = 100 min. The highest average temperature (302.45 K) occurred at the lowest flow rate at t = 100 min, while the lowest average panel temperature was observed to be 300.63 K at t = 100 min. The energy storage of the PCM had a strong contribution to the average panel temperature and improved the temperature uniformity on the panel, enabling higher temperature control and minimizing sharp rises or drops in the temperature upon changes in the boundary conditions.

**Figure 6:**
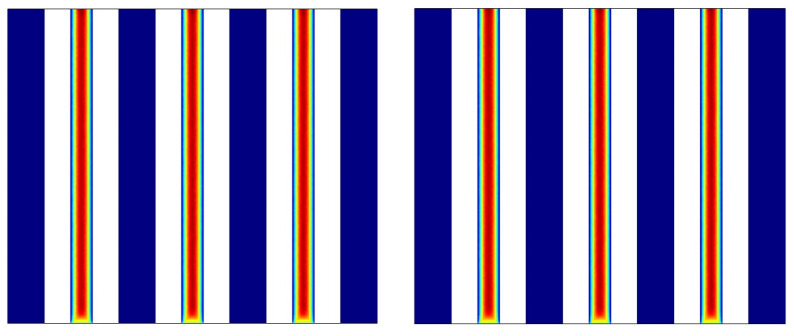

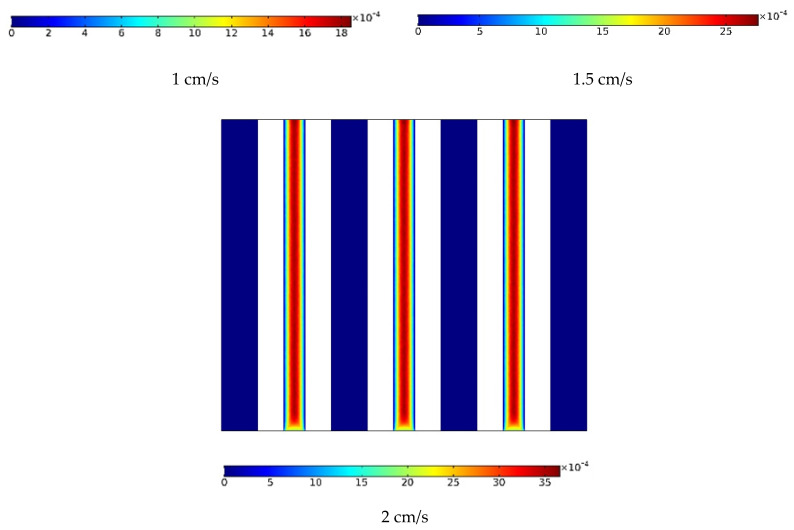
The velocity in the horizontal middle section at three velocities.

Figure 6 indicates the velocity in the horizontal middle section at three velocities. A rise in the input velocity (flow rate) influenced the maximum velocity in the middle of the pipes. In addition, the average velocity increased as the input velocity increased. The velocity was almost zero in the PCM and around fins due to their rigidity. Due to the no-slip boundary condition, the velocity was low near the pipe wall and higher near the centerline. The flow velocity was maximized in the middle of the pipe. An increased input velocity may enhance the heat discharge through the flow. 

**Figure 7:**
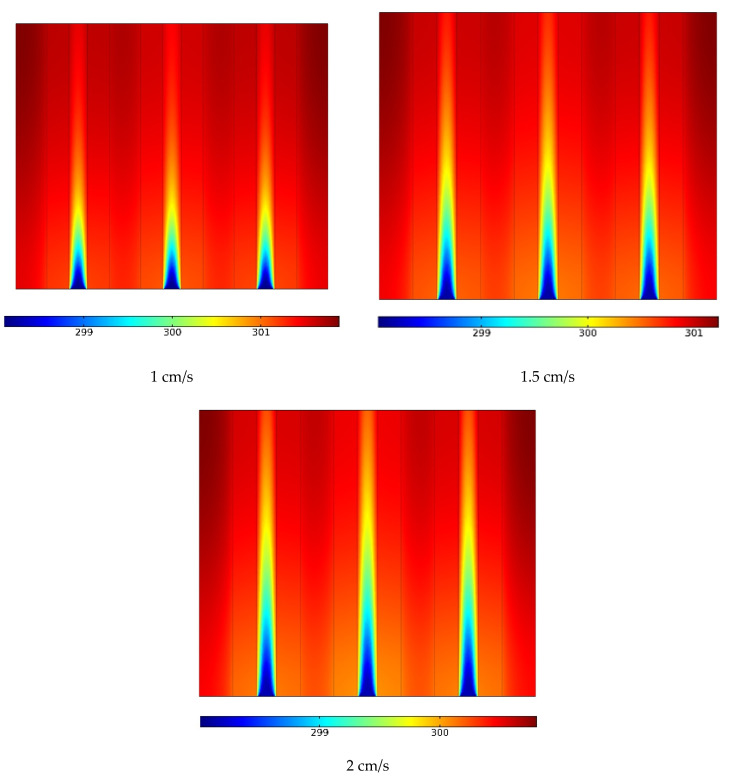
The temperature contour at three different velocities in the solar panel’s horizontal surface.

Figure 7 shows the temperature contour at three different velocities in the center of the solar panel’s horizontal surface. The temperature of the panel was considerably lowered by an increase in velocity (flow rate). As the flow rate increased, the maximum panel temperature decreased. Due to the PCM passive temperature control system, the panel temperature had a small dependence on the flow rate; the dependence of the temperature on the flow rate could be much greater in the absence of the PCM. The increased flow rate led to a lower NF temperature along the pipe, with a larger area of the panel being in contact with a cooler flow. At the lowest flow rate, the flow temperature was similar to the PCM temperature at the beginning of the pipe; as a result, the panel had a higher temperature at longer times on the output side.

**Figure 8:**
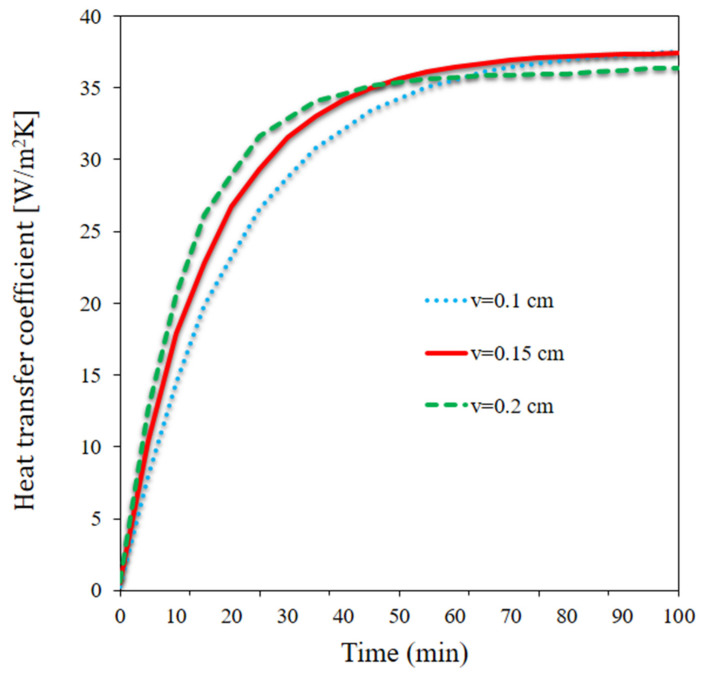
The HTR coefficient between the pipes and panel at three velocities over time.

Figure 8 shows the HTR coefficient between the pipes and panel at three velocities over time. The HTR coefficient increased from t = 0 to t = 80 and 90 min, regardless of the flow rate. It initially rose at a greater rate. The HTR variation was small at longer times and remained almost unchanged after a certain period. The HTR coefficient was higher at one of the three flow rates. It was maximized at the highest flow rate at t = 0 to t = 40 min, whereas it was maximized at the medium flow rate at times longer than 40 min. The lowest flow rate led to the maximum heat coefficient at final times. When we first started measuring, there was a larger temperature gap between the panel and the NF. Because of the lower temperature of the flow, the HTR coefficient was increased, particularly in the beginning, while the heat from the sun melted the PCM and caused the temperature of the PCM to rise. The maximum flow rate effect on the HTR coefficient occurred from t = 10 to t = 40 min; the melted PCM difference at different flow rates led to different HTR coefficients.

**Figure 9:**
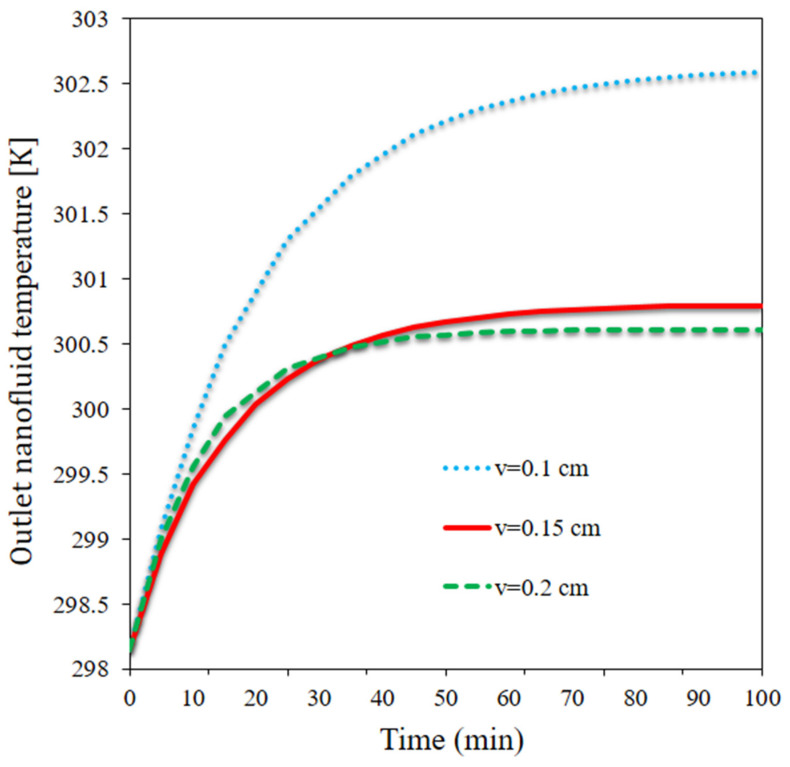
The output temperature at three velocities.

Figure 9 plots the output temperature at three velocities. The output temperature of the flow is strongly dependent on the flow rate. The output temperature consistently increased at the lower flow rate (initially a large rate and then a moderate rate). At the medium and higher flow rate, however, the output temperature initially increased and then remained almost unchanged after a certain time. Between t = 0 and t = 30 min, the output temperature was greater when the flow rate was higher; however, after t = 30 min, the output temperature was higher when the flow rate was medium. Over the course of time, the greatest output temperature occurred at the lowest flow rate. The PCM energy storage influenced the output temperature. The output temperature decreased by nearly 2 °C as the flow rate doubled at t = 100 min. 

**Figure 10:**
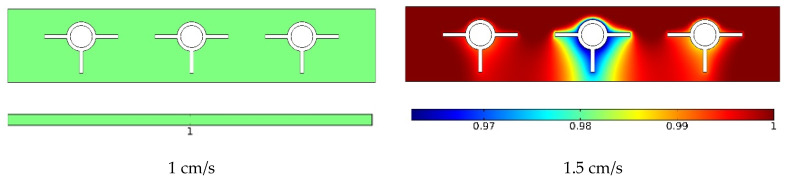

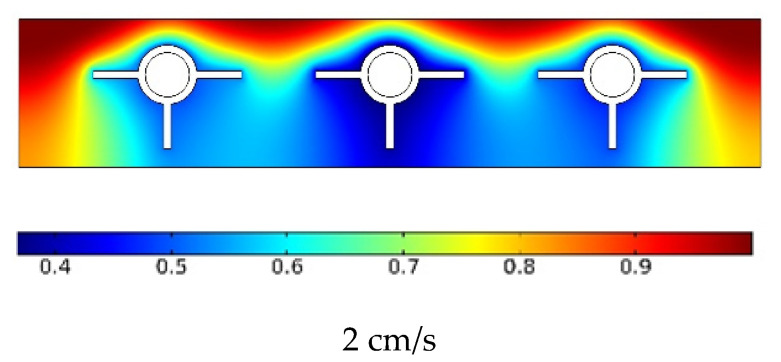
The melted PCM volume fraction in the vertical middle section at three velocities.

In the center of the vertical portion at three velocities, Figure 10 shows the volume percentage of melted PCM. The phase shift of the PCM was impacted by the flow rate in the pipes. Because of the sun’s heat, the PCM rapidly melted in its entirety despite the decreased flow rate. When the flow rate was increased to medium, a bigger proportion of the PCM melted, with a minuscule quantity of the PCM remaining non-melted in the region around the pipes and fins. The PCM largely melted near the top of the PCM cavity when the flow rate was greater; however, a larger section of the PCM did not melt when the flow rate was higher. In particular, the PCM delivered heat to the NF in the vicinity of the pipes, diminishing the phase change energy and the melted PCM fraction. The non-melted PCM fraction was greater around the middle pipe due to the two lateral other pipes. The lateral pipes were insulated on one side, leading to HTR in the PCM and a phase change into a melt. 

**Figure 11:**
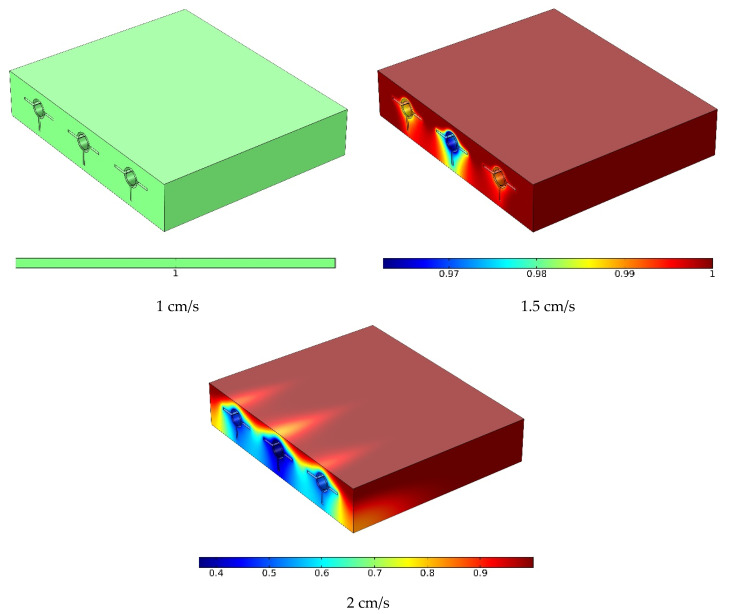
The melted PCM volume fraction contour around the panel at three velocities.

Figure 11 depicts the curve of the melted PCM volume percentage at three different velocities around the panel. At the lower flow rate, the entire PCM melted in 10 min. At a velocity of 0.1 m/s, the major portion of the PCM melted, with a small fraction remaining non-melted around the middle pipe. At the higher flow rate, however, the PCM mostly remained non-melted in the middle, while the outer areas of the PCM melted. These regions absorbed more solar heat and gave out less heat to the water flowing through the pipes. On the other hand, a lower amount of heat was used in the PCM’s phase transition as a result of increased HTR from the PCM to the middle pipes. Moreover, the PCM remained non-melted at the top of the outer PCM cavity layer on the input flow side, while the PCM on the output side entirely melted.

**Figure 12:**
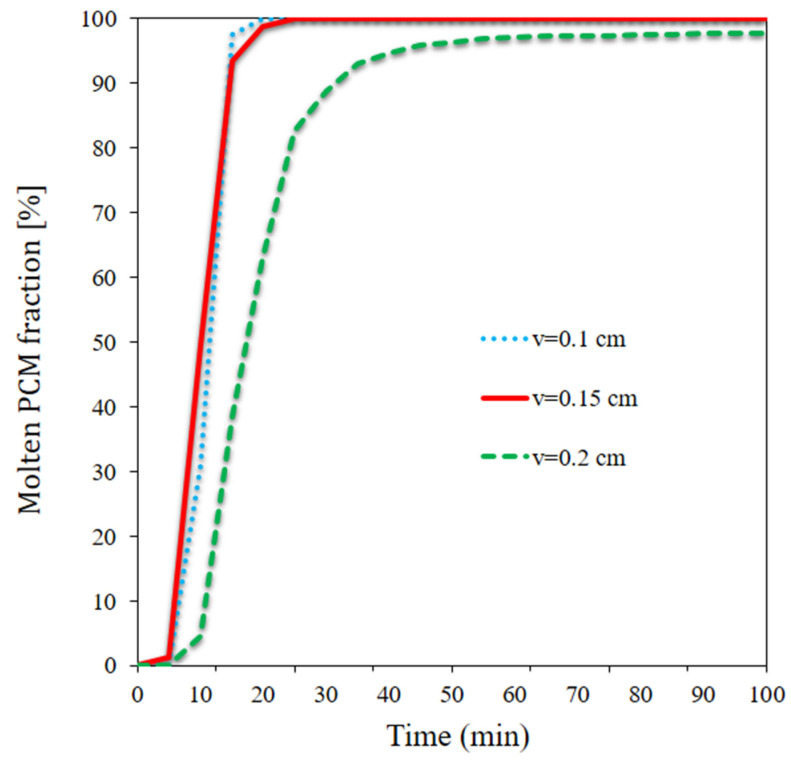
The melted PCM volume fraction for three velocities over time.

Figure 12 depicts the melted PCM volume fraction for three velocities over time. The melted PCM volume fraction is dependent on solar radiation and HTR between the PCM and NF flow. Higher solar radiation injects a greater quantity of heat to the PCM and accelerates PCM melting. By absorbing more heat from the PCM, a higher flow velocity slows down PCM melting. As the solar heat flux was high, the PCM entirely melted in a short time at two flow velocities. A change in flow velocity (flow rate) had a negligible impact on PCM melting in the solar system because the heat discharge was weak at the lower flow velocity. Regardless of the flow velocity, the PCM started to melt before t = 10 min, and depending on the flow velocity, a significant portion of the PCM melted. However, the PCM did not completely melt at 0.2 cm/s; by t = 50 min, the melted PCM portion was above 90%. The PCM melting rate substantially declined after t = 60 min, with the system becoming stabilized. 

## 5. Conclusions

In this study, we simulated NF flow within a solar system consisting of a solar panel and a PCM at three NF velocities. The two-phase mixture was adopted to simulate the NF. Three finned pipes were placed within the PCM to reduce the temperature and improve temperature uniformity. The PCM and NF were organic and eco-friendly. The system was simulated in a transient state. The results can be summarized as follows:

1. The PCM entirely melted in a short time at the lower and medium flow rates; at the higher flow rate, however, a portion of the PCM remained non-melted.
2. The output temperature decreased by nearly 20 °C at t = 100 min as the input velocity (flow rate) doubled.
3. The HTR coefficient was maximized at one of the flow rates; it was initially maximized at the higher flow rate, whereas the maximum HTR coefficient occurred at the medium flow rate at longer times.
4. The average temperature of the panel declined by 1.82 °C at t = 100 min as the flow rate doubled. The highest average temperature was 302.45 K and occurred at the lower flow rate at t = 100, while the lowest average temperature was observed to be 300.63 K and occurred at the highest flow rate.
5. The maximum temperature of the panel reduced by 1.85 °C at t = 100 min as the flow rate doubled.

## 6. Challenges and Prospects

Some suggestions are given for future work according to the study conducted in the field.

1. The impact of changing the use of these materials on the efficiency of the solar panel can be examined by changing the type of nanofluid and PCM.
2. It is possible to assess the performance of the solar system during liquid-to-solid phase change to examine the thermal efficiency of the solar panel.
3. The effect of using fins with different shapes and different numbers in PCM can be considered.

## Figures and Tables

**Table 1:** PCM properties [48].

PCMs Type	Melting Point (°C)	$\mathrm{Heat}\;\mathrm{Latent}\;\left(\frac{J}{g}\right)$	$\mathrm{Thermal}\;\mathrm{Conductivity}\;\left(\frac{W}{mK}\right)$
Paraffin wax/nanoegraphite	27.73	202.1	0.365

**Table 2:** Organic nanoparticle properties [49].

	*d_s_* (mn)	μ (kg/m·s)	ρ (kg/m^3^)	*c_p_* (kg·k)	*k* (W/m·K)
Water	-	0.001	997.1	4179	0.613
EG	-	0.0141	1088	2430.8	0.2532
Ag	40	-	10500	235	429

**Table 3:** Average panel temperature in different grids for a velocity of 0.1 cm/s.

Element number	908,035	1,013,730	1,144,853	1,291,256
V-Max (m/s)	0.0020	0.0019	0.0018	0.0018
T-mid (K)	300.38	300.61	300.74	300.74

**Table 4:** The numerical model and Bizhaem and Abbassi [53] in the pressure drop at the Reynolds numbers of 200, 500, and 1000 for volume fractions of 1% and 3%.

Re	200	500	1000
φ = 1%			
Ref. [53]	5.56	5.31	5.17
This paper	5.42	5.19	4.98
%Err	2.25	2.2	3.6
φ = 3%			
Ref. [53]	1.77	1.65	1.60
This paper	1.71	1.60	1.52
%Err	3.4	3.0	5.0

**Table 5:** Outlet temperature values of water at different flow rates in a solar collector at different hours: a comparison between the present work and the experimental data of Aghakhani et al. [54].

Flow Rate	0.5	1.5	2.5
Ref. [54]	304.26 K	301.86 K	296.50 K
This paper	304.68 K	301.67 K	296.11 K
Difference	0.42 K	0.19 K	0.39 K

## Data Availability

Not applicable.

## Nomenclature

C	Mushy constant	**Greek symbols**	
Cp	Specific heat [J/(kg.K)]	α	Thermal diffusivity $\left[m^{2}/s\right]$
F	Faraday’s constant	φ	Volume fraction
Fi	Volume fraction	μ	Dynamic viscosity [w/(m.K)]
g	Gravitational acceleration [m/s^2^]	ρ	Density $\left[\mathrm{Kg}/m^{3}\right]$
H	Enthalpy [kJ/kg]		
k	Thermal conductivity [W/(m.K)]		
p	Pressure [Pa]	**Subscripts**	
PCM	Phase change material	bf	Pure fluid
Re	Reynolds number	dr	Drift
t	Time (s)	HRT	Heat transfer rate
T	Temperature [K]	m	Mixture
u, v, w	Velocity components [m/s]	nfs	Nanofluid
x, y, z	Cartesian coordinates [m]	pf	Solid nanoparticle
